# Age-Associated B Cells in Autoimmune Diseases: Pathogenesis and Clinical Implications

**DOI:** 10.1007/s12016-025-09021-w

**Published:** 2025-02-17

**Authors:** Guangyang Xie, Xiaojing Chen, Yixia Gao, Ming Yang, Suqing Zhou, Liwei Lu, Haijing Wu, Qianjin Lu

**Affiliations:** 1 Department of Dermatology, the Second Xiangya Hospital, Hunan Key Laboratory of Medical Epigenomics, Central South University, Changsha, Hunan China; 2 Department of Pathology and Shenzhen Institute of Research and Innovation, The University of Hong Kong, Hong Kong, China; 3 Centre for Oncology and Immunology, Hong Kong Science Park, Hong Kong, China; 4 Institute of Dermatology, Chinese Academy of Medical Sciences and Peking Union Medical College, Nanjing, Jiangsu China; 5 FuRong Laboratory, Changsha, China

**Keywords:** B-lymphocytes, Aging, Autoimmune diseases, Pathogenesis

## Abstract

As a heterogeneous B cell subset, age-associated B cells (ABCs) exhibit distinct transcription profiles, extrafollicular differentiation processes, and multiple functions in autoimmunity. TLR7 and TLR9 signals, along with IFN-γ and IL-21 stimulation, are both essential for ABC differentiation, which is also regulated by chemokine receptors including CXCR3 and CCR2 and integrins including CD11b and CD11c. Given their functions in antigen uptake and presentation, autoantibody and proinflammatory cytokine secretion, and T helper cell activation, ABCs display potential in the prognosis, diagnosis, and therapy for autoimmune diseases, including systemic lupus erythematosus, rheumatoid arthritis, Sjögren’s syndrome, multiple sclerosis, neuromyelitis optica spectrum disorders, and ankylosing spondylitis. Specifically targeting ABCs by inhibiting T-bet and CD11c and activating CD11b and ARA2 represents potential therapeutic strategies for SLE and RA. Although single-cell sequencing technologies have recently revealed the heterogeneous characteristics of ABCs, further investigations to explore and validate ABC-target therapies are still warranted.

## Introduction

Age-associated B cell (ABC) subset is identified as a heterogeneous B cell subpopulation [1]. ABCs exhibit unique transcriptional profiles and functional properties characterized by expressing T-bet (T-bet positive), CD11c, and CD11b, along with a low expression level of CD21 [2]. ABCs respond to the antigen-induced inflammatory and immune microenvironment through various signaling pathways, including toll-like receptors (TLRs) and cytokines [3]. Unlike follicular B cells, B cell receptor (BCR) signaling alone is insufficient to induce ABC proliferation but can coordinate with TLR7/9 signaling to initiate downstream pathways [4]. T-bet expression in ABCs is regulated by cytokines such as IFN-γ and IL-21 [5]. Various B cell subsets contribute to the ABC pool, including naïve B cells, follicular B cells, marginal zone (MZ) B cells, and transitional B cells. Regardless of the origin of their precursor cells, ABCs differentiate into antibody-secreting cells (ASC) and memory B cells (MBCs), thereby maintaining the humoral immunity balance [4, 6].

Autoimmune diseases result from autoreactive adaptive immune response [7]. Over the past decades, the prevalence of autoimmune diseases has been rising, posing huge burdens to public health [8]. The pathogenic role of ABCs is crucial in the development of autoimmune diseases, including systemic lupus erythematosus (SLE) [9, 10], rheumatoid arthritis (RA) [10, 11], Sjögren’s syndrome (SS), multiple sclerosis (MS) [10, 12], neuromyelitis optica spectrum disorders (NMOSD), and ankylosing spondylitis (AS), through autoantibody and proinflammatory cytokine secretion, enhanced antigen presentation, and follicular T helper cell (Tfh) activation [13]. For example, ABCs are regarded as significant precursors of ASCs in SLE and ABCs have been reported to produce low levels of antibodies upon certain stimulation [1, 14, 15]. ABCs are also recognized as the major B cell subsets in synovial fluid of RA patients [16] and interact with synovial-like fibroblasts (FLS) in RA pathogenesis [11]. Therefore, ABCs represent the potential as a new target for autoimmune diseases.

This review describes the development process of ABCs and reveals the essential signaling pathways in ABC differentiation, including TLR7 and TLR9 signals, IFN-γ and IL-21 signals, chemokines, and integrins. Moreover, we summarize research advances in understanding the role of ABCs in autoimmunity and autoimmune diseases and explore the potential of ABCs in the prognosis, diagnosis, and therapy for autoimmune diseases. More studies on ABC-specific therapies for SLE and RA, such as T-bet and CD11b/c modulators, are still warranted.

## The Development Process of ABCs

**Fig. 1:**
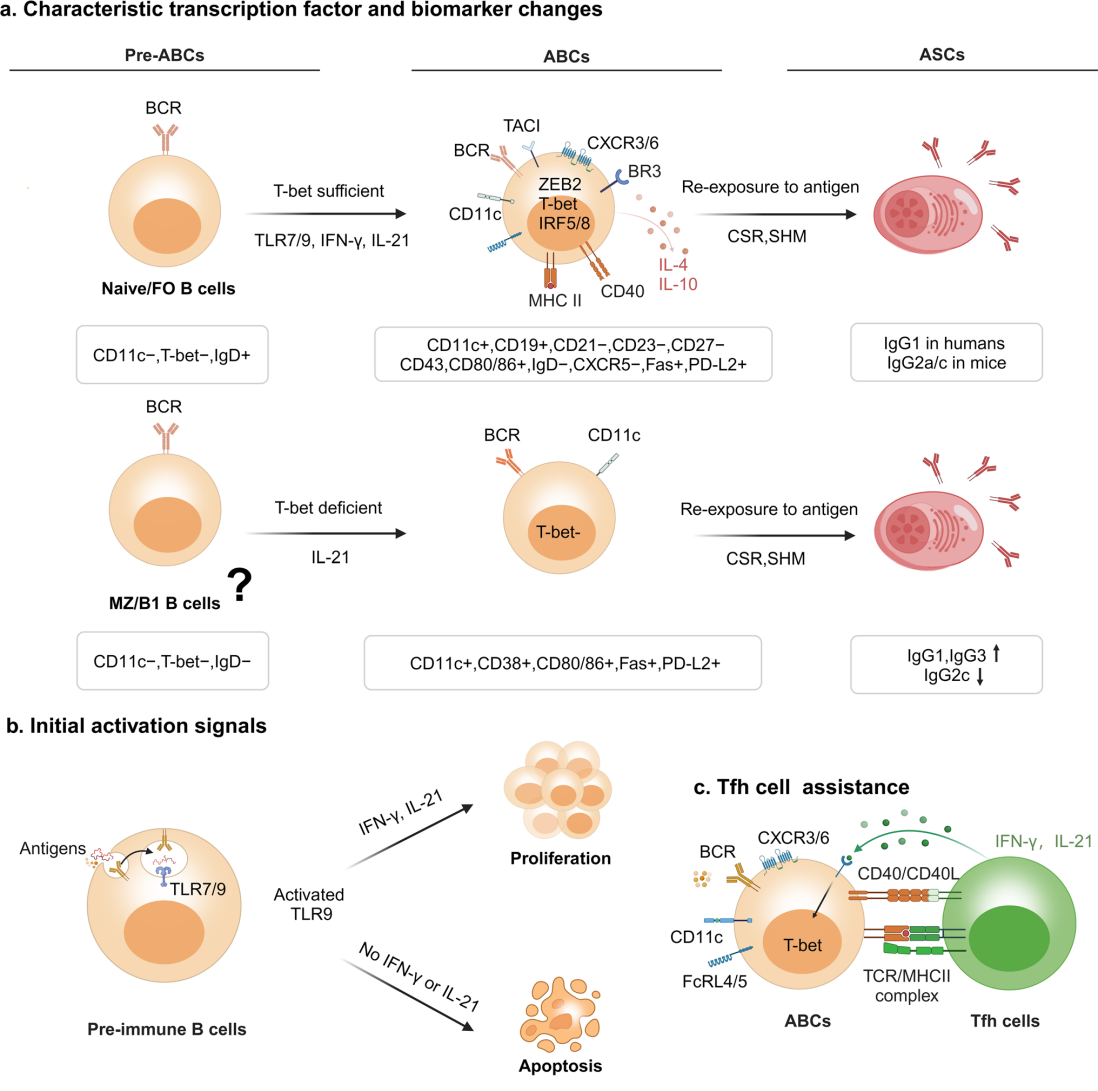
The development process of ABCs. ASC, antibody-secreting cell; FcRL5, Fc receptor-like protein 5; FO B cells, follicular B cells; MZ B, marginal zone B cell; SHM, somatic hypermutation; Tfh, follicular T helper cell. (Created with biorender.com)

Researchers have been actively investigating the origins of ABCs and have identified certain B cell subsets as their potential progenitors. It has been reported that ABCs can differentiate from peripheral B cells [13]. Furthermore, naive B cells from healthy individuals can be successfully induced to differentiate into ABCs in vitro [6]. Hao et al. concluded that young follicular B cells have the potential to proliferate and generate ABCs extensively [4]. Moreover, the innate sensor signal during B cell differentiation, which often results in the heterogeneous generation of autoreactive or multireactive antibodies [17], suggests that other B cell subsets, such as transitional, marginal zone, or B1 cells, may also constitute the ABC pools (Fig. 1a).

When exogenous antigens invade or endogenous antigens emerge, these antigens bind to BCRs on B cells, leading to antigen endocytosis. Simultaneously, the innate sensors TLR7/9 are activated, triggering the development shift from pre-immune B cells to ASCs [18]. Crucially, TLR9 activation without subsequent signals or interactions prompts pre-immune B cells to undergo apoptosis, contributing to inherent peripheral tolerance [19]. On the contrary, when pre-immune B cells receive stimulation from IFN-γ and IL-21 signals, they undergo profound alterations in expressing transcription factors, chemokine receptors, FcRLs, etc. Specifically, elevated levels of T-bet, HLA-DR, and co-stimulatory molecules are also observed [16] (Fig. 1b).

T-bet-expressing B cells differentiate beyond the follicle, facilitated by homologous T-cell interactions and the CD40/CD40L engagement. They ultimately showcase a wide range of *VH* and *Vκ* genes featuring somatic hypermutations (SHM), giving rise to plasmablasts and T-bet antibody-producing MBCs [20]. Upon encountering secondary antigens, these cells swiftly differentiate into ASCs, producing class-switched IgG antibodies, primarily IgG1 in humans and IgG2a/c in mice [21]. Similarly, naive B cells are inclined to quickly differentiate into ASCs upon stimulation with cytokines IFN-γ, IL-21, and TLR7/9 activation in vitro (Fig. 1c).

Unlike other B cell subpopulations, ABCs may possess unique activation and proliferation requirements. ABCs do not initiate proliferation when it comes to the BCR stimulation alone in vitro, but they rely on the transduction of endosomal TLR signals, especially from TLR7 and TLR9. In acute lymphocytic choriomeningitis virus (LCMV)-infected mice, STAT1 signaling is a key driver of ABC activation and proliferation [22]. Additionally, IL-21 promotes autoreactive T-bet CD11c^high^ B cell expansion in SLE, potentially through STAT1-dependent signaling pathways [5]. However, the precise regulatory mechanisms of the ABC development and fate determination require further investigation.

## Signaling Pathways in ABC Differentiation

**Fig. 2:**
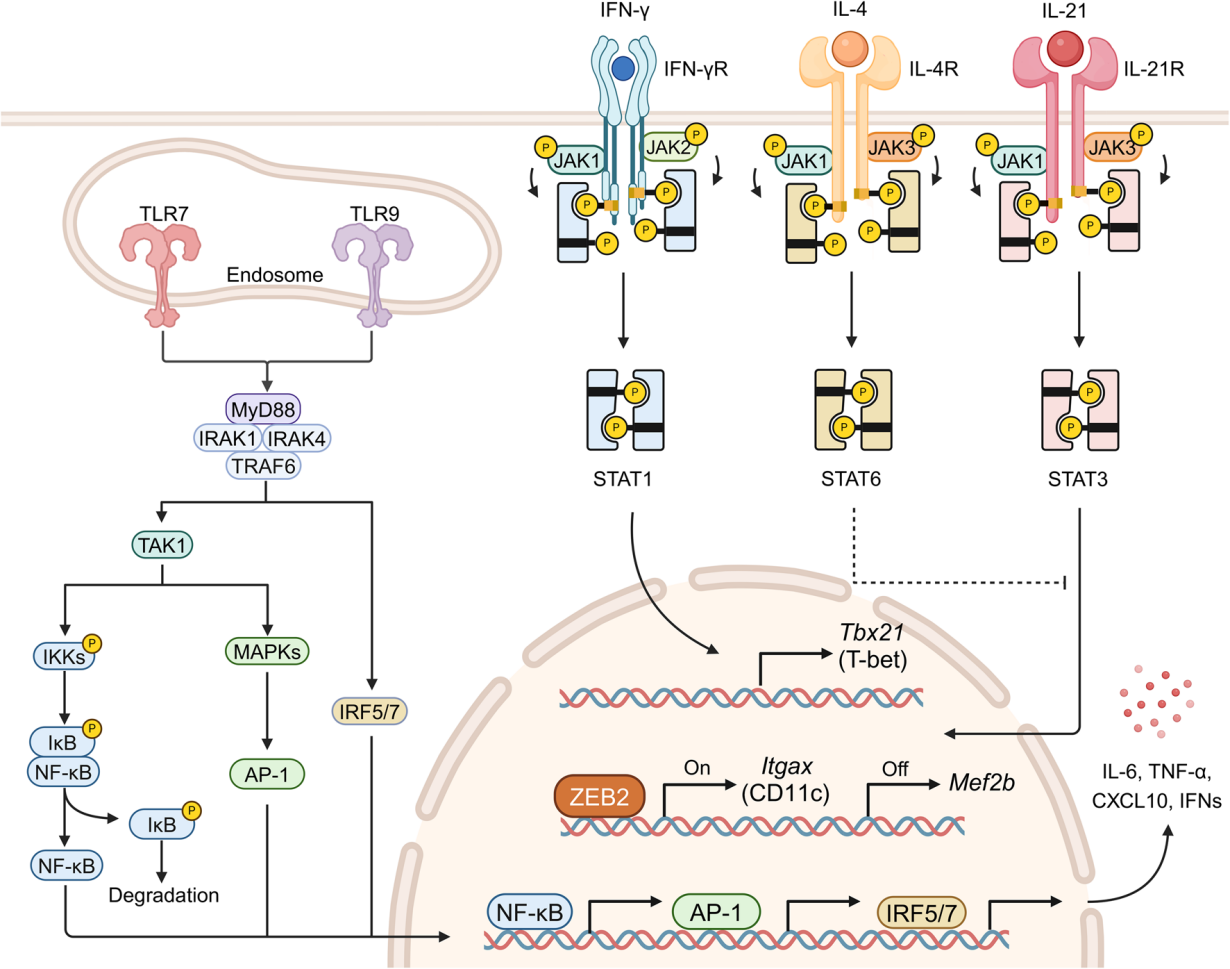
Signaling pathways in ABC differentiation. Itgax, integrin subunit alpha X; Mef2b, myocyte enhancer factor 2b; Tbx21, T-box protein 21. (Created with biorender.com)

The differentiation process and fate determination of ABCs are complicated and regulated by multiple signal pathways induced by TLRs, cytokines, chemokine receptors, and integrins (Fig. 2). All of them are integral to ABC development, contributing to antigen recognition, phenotype determination, cellular migration and adhesion, and Tfh interaction.

First and foremost, TLR signaling is pivotal in B cell differentiation and activation [23]. Upon activation, TLRs trigger NF-κB, AP-1, and IRFs, mobilizing the MyD88 or TRIF pathway to mediate downstream signaling, producing inflammatory cytokines and interferons that activate T and B cells [24]. Notably, TLR7/9 play a vital role in ABC expansion and ASC differentiation, thus promoting autoimmune diseases [25, 26]. TLR7 functions independently of the classical IFN-α/β pathway, requiring MyD88 for ABC development [27]. Activated MyD88 and NF-κB signaling initiates the transcription of proinflammatory cytokines and may cause activation-induced cytidine deaminase, contributing to SHM and class-switch recombination in ABCs [28]. TLR9 synergized with BCR to stimulate antigen-specific B cells [29]. TLR9 ligand led to a vigorous proliferation of ABCs [4]. The role of TLR9 signaling is MyD88-independent in lupus, while the MyD88-mediated proinflammatory signaling promotes disease [27]. However, the intricate mechanisms and cytokine interactions require further elucidation in TLR7/9-induced ABC differentiation.

Apart from TLR7/9 agonism, the involvement of IFN-γ and IL-21 in ABC differentiation is essential for inducing ABC expansion in vitro. IFN-γ and BCR signals are most important in the initial phase, while IL-21 is most needed in the late amplification and differentiation phase, and the TLR7 stimulation is vital throughout the culture period. Early IFN-γ stimulation enhanced IL-21R signaling, allowing cells to differentiate smoothly even under the suboptimal TLR7 stimulation [6]. IFN-γ levels in ABCs were significantly higher in patients with acquired aplastic anemia, indicating a vicious cycle of IFN-γ production and ABC generation [30]. Intriguingly, IL-21 and TLR7/9 co-stimulation was sufficient to induce ABC differentiation and cutaneous lupus development [15]. IL-21 and IRF5 enhanced the generation of ABCs in lupus-associated SWEF-deficient mice [31].

The complex cytokine network, primarily involving Tfh cell-secreted IL-21 [32], IFN-γ [5], and IL-4, intricately mediates T-bet expression in ABC differentiation. In response to Th1 triggered by viral infections or nucleic acid antigens, the secreted cytokines IFN-γ and IL-21 would jointly promote T-bet expression in the presence of TLRs [32]. IL-21-induced T-bet expression was activated only by TLR7 or TLR9 in vitro B cells. Notably, IFN-γ upregulates the expression of T-bet mainly through STAT1, while IL-21 activates STAT1/3/5 to promote T-bet and CD11c expression [5, 33]. A systemic level of IFN-γ was upregulated in concert with an elevated level of T-bet B cells, mediated by the interaction of endogenous retroviruses with TLRs [18]. IFN-γ and its downstream T-bet signaling regulated aerobic glycolysis metabolism in ABCs, thereby contributing to their development and differentiation [34]. IL-4 plays a complex role in enhancing IFN-γ-induced but inhibiting IL-21-induced T-bet expression depending on STAT6 [32]. These findings indicated that T-bet expression is comprehensively determined by the relative levels of IL-21, IFN-γ, and IL-4 [33].

Meanwhile, chemokine receptors regulate the migration and localization of ABCs. CXCR3, CCR6, CCR7, and CCR9 upregulation enhanced the migration of ABCs to the inflamed sites [35, 36], while the expressions of CXCR4, CXCR5 (follicle homing factor), and CCR7 decreased [37]. ABCs migrated to the T-cell region and eventually accumulated at the splenic T-B boundary, which was also evidenced by the enhanced reactivity of ABCs to CCL21 and CCL19 in the T-cell region [38]. However, CXCR3 was downregulated in ABCs from the peripheral blood of patients with IgG4-related disease [39].

During migration and localization, the upregulation of integrins is significant for ABC adhesion. T helper cell assistance and ABC antigen presentation require intercellular contact, where integrins are important. After the resolution of infection, T-bet CD11c ABCs remained in the splenic marginal region with elevated expression levels of integrins, including LFA-1 (αLβ2, encoded by *Itgal* and *Itgb2*, respectively) and VLA-4 (α4β1, encoded by* Itga4* and *Itgb1*, respectively) [22].

## Dysfunction of ABCs in Autoimmunity

With unique surface markers, ABCs hold the features of myeloid-derived cells and classical B cells [40]. ABCs possess diverse functions, including phagocytosis, antibody secretion, proinflammatory cytokines, chemokine release, antigen presentation, and T helper cell activation. Thus, ABCs are crucial in mounting an effective immune response against different infectious agents in humoral immunity [41]. However, excessive proliferation and accumulation of ABCs have detrimental effects, contributing to autoinflammatory and autoimmune diseases, for example, SLE and RA. Therefore, maintaining a balanced immune response is paramount to preventing such pathological outcomes.

ABCs play a critical role in orchestrating autoimmunity and inflammation. ABCs can quickly differentiate into ASCs stimulated by pathogens or IL-21 and IFN-γ signals, as well as TLR7/9 signals. This differentiation process results in the secretion of IgM and IgG2a/c isotypes. The IgG2a/c isotype is associated with protective antiviral responses and pathogenic autoantibodies (such as anti-dsDNA and anti-Smith autoantibodies in lupus mice). In lupus-like autoimmunity, autoreactive ABCs are recruited to the inflamed site and subsequently produce IgG2a, IgG2b, and IgG3, exacerbating persistent inflammation. In addition, ABCs strongly correlate with antiphospholipid antibodies (such as anti-phosphatidylserine and anti-cardiolipin antibodies) under infection and autoimmune conditions [40]. Besides, consistent with myeloid-like characteristics, ABCs exhibit an enhanced ability to generate proinflammatory cytokines and chemokines, including IL-6, IFN-γ, CXCL10, and CCL5, different from other B cell subpopulations. They possess a distinctive and characteristic repertoire of inflammatory and regulatory cytokines, featured by elevated levels of IFN-γ and IL-10 [42–44]. The transcriptome data of ABCs from the peripheral blood of patients with early RA differs significantly from those of healthy controls, marked by increased expression of chemokine receptors and adhesion molecules, for example, CXCR3, which facilitates the migration of ABCs to the inflamed sites [16].

ABCs are highly proficient antigen-presenting cells with strong phagocytic capacity. In lupus-prone *Ldlr-/-* mice, ABCs enhanced the expression of genes associated with antigen presentation [45]. ABCs exhibit unique phagocytic properties and efficient antigen phagocytosis and hold a robust phagocytic capacity to uptake apoptotic cells in vitro. Additionally, ABCs are enriched with signaling pathways related to phagocytosis, including phagosome formation, FcγR-mediated phagocytosis, and FcεR-1 signaling. Interestingly, ABCs also express genes associated with cytotoxicity like NKG7, granzyme A, and perforin [2, 5], which may contribute to the overall efficiency and effectiveness of antigen presentation. ABCs can skew initial CD4 T-cell differentiation towards the Th17 lineage when functioning as antigen-presenting cells in vitro [3]. Furthermore, the impact of ABCs on activating inflammatory T cells can be observed in certain autoimmune diseases. For instance, coculturing ABC-like cells from Crohn's disease patients with autologous CD4 T cells promoted T cells to generate IFN-γ and IL-12 [46]. T-cell deficiencies were evident in mice with SLE lacking ABC subsets, with significantly reduced numbers of activated memory CD4 T cells and IFN-γ-producing CD8 T cells in contrast to mice with intact ABC populations [47].

## ABCs in Autoimmune Diseases

**Fig. 3:**
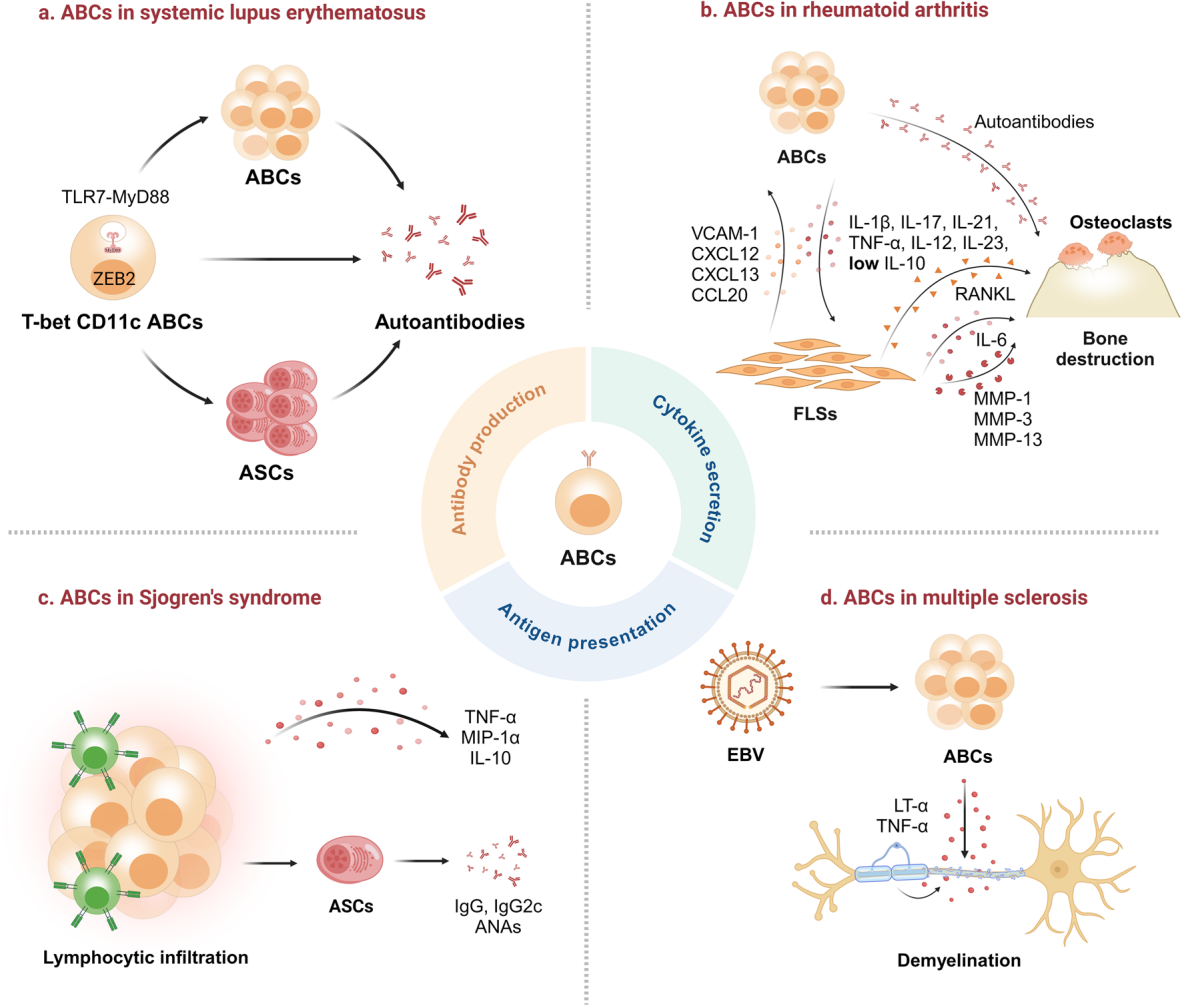
The pathogenic role of ABCs in some autoimmune diseases. ANA, antinuclear autoantibody; FLSs, synovial-like fibroblasts; LT-α, lymphotoxin-α; MIP-1α, macrophage inflammatory protein-1α; MMP, matrix metalloproteinase; RANKL, receptor activator of the NF-κB ligand; VCAM-1, vascular cell adhesion molecule 1. (Created with biorender.com)

The pathogenic role of ABCs is crucial in autoimmune diseases, especially in SLE and RA [9–13]. ABCs can produce autoantibodies, promote inflammatory responses, and stimulate Tfh differentiation, all contributing to autoimmune diseases (Fig. 3). TLR7 and TLR9 signals, IL-21, and IFN-γ are also essential for ABC differentiation. These findings suggest that ABCs represent novel targets for diagnosing and managing autoimmune diseases. However, exploring potential therapeutic interventions remains challenging until the specific functions and mechanisms underlying ABCs and autoimmune diseases are fully understood (Table 1).

**Table 1:** ABCs in autoimmune diseases

Subjects	Markers	Sample types	Dysfunctions of ABCs	Ref
***SLE***				
SLE patients, lupus mice	T-bet, CD11c, CD19, CD21 −	Peripheral blood	ABCs accumulated in lupus patients and mice and correlated with IgG levels and SLE clinical phenotypes and prognosis	[50, 65–67]
Lupus nephritis patients	ABC-expressed genes	Kidney	Naïve B cells were activated to ABCs in the kidney, probably driven by BCR/TLR ligands	[68]
SLE patients	IgD − , CD27 − , CD11c, CD21 −	Peripheral blood	Rituximab treatment reduced ABC frequency during early follow-up	[71]
SLC^−/−^ and MRL/lpr mice	CD19, CD21 − , CD23 − , CD43 − , CD93 −	Spleen	ABCs expressed MBC markers, underwent SHM, and secreted typical lupus autoantibodies such as anti-Smith antibodies	[52]
MRL/lpr mice	CD11b/c, T-bet	Spleen	ABCs were proliferative and underwent self-renewal, giving rise to plasmablasts and GC B cells, contributing to renal diseases	[1]
Imiquimod-induced, bm12-induced, and LCMV infection lupus mice	CD11c, CD19, IgD −	Spleen	ZEB2 drove human and mouse ABC differentiation and the dissemination to inflamed sites, as well as producing antinuclear antibodies and proinflammatory cytokine and chemokine	[144]
BXD2 lupus mice	T-bet, CD11c, IgD −	Spleen	T-bet CD11c ABCs caused the female-predominant production of autoantibodies	[53]
*Def6/Swap70* DKO mice	B220, CD19, CD11b/c	Spleen	ABC accumulation and function were sex- and TLR7-dependent. TLR7 duplication promoted sex bias and lupus development by enhancing ABC accumulation and autoantibody production	[54]
TLR7^Y264H^ patients and mice	B220, CD21 − , CXCR5 − , CD19, CD11c	Spleen, peripheral blood	Enhanced TLR7 signaling promoted the aberrant survival of active B cells and the accumulation of CD11c ABCs and GC B cells, which could be rescued by MyD88 deficiency	[26]
TLR9^K51E^ and TLR9^P915H^ MRL/lpr mice	TCRβ − , CD11b/c, CD19	Spleen	TLR9 controlled ABC gene expression independently of MyD88	[27]
*Def6*/*Swap70* DKO, IL-13Rα1-KO mice	CD11b/c, T-bet	Spleen	IL-13Rα1 was elevated in ABCs and the deletion of IL-13Rα1 suppressed ABC accumulation and their differentiation into plasmablasts	[58]
Humanized CLE mice	T-bet, CD11b	Spleen	IL-21 stimulation and TLR signals prompted ABC proliferation, differentiation, and IgG secretion	[15]
*Def6*/*Swap70* DKO, *Irf5* DKO mice	B220, CD19, CD11b/c, T-bet	Spleen	ABCs spontaneously expanded, and IL-21-mediated ABC generation was dependent on IRF5. SWEF proteins modulated the proliferation and proinflammation of ABCs	[31]
Bm12-induced lupus mice	T-bet, CD11c	Spleen	IFN-γ signaling contributed to the increased glycolytic capacity of ABCs and glycolysis restriction could inhibit ABC formation. T-bet mediated glycolysis pathway genes by inhibiting *Bcl6* expression	[34]
Ship-deficient lupus mice	T-bet, CD11c	Spleen	Excessive ABCs coincided with dysregulated Tfh cells. Overabundant ABCs triggered Tfh differentiation through antigen presentation and immunological selection. MyD88 ablation blocked ABC differentiation and rescued the subsequent drawbacks	[59]
Sle1/2/3 triple-congenic mice	T-bet, CD11c, CD21 − , CD23 −	Spleen	ABCs induced stronger TCR signaling than follicular B cells and preferentially promoted Tfh differentiation in an antigen-dependent manner. Metformin treatment suppressed antigen uptake by ABCs and reduced Tfh differentiation	[60]
SLE patients	T-bet, CD11c	Peripheral blood	RNase2 was elevated in SLE patients in SLE patients and highly expressed in their PBMCs. RNase 2 induced the IL-10 expression in monocytes to promote ABC formation	[61]
Imiquimod-induced lupus and aged *Fcrl5* transgenic mice	CD19, B220, CD43 − , CD23 − , CD21 − , CD11b/c	Spleen	FcRL5 was highly expressed in ABCs, and FcRL5 overexpression exacerbated systemic autoimmunity with age, as FcRL5 upregulation facilitated TLR signaling and destroyed B cell anergy	[62]
SLE patients	CD11c, CD27 − , IgD −	Peripheral blood	A reduction in P2RY8 was associated with increased ABCs and ASCs and deteriorated lupus nephritis	[63]
SLE patients	CD11c, CD21 −	Peripheral blood	Myeloperoxidase-DNA complexes were positively associated with the percentages of ABCs and MBCs	[64]
***RA***				
Male C57B6/L mice, RA patients	B220, CD11c, T-bet in mice; CD19, CD27 − , IgD − , CD21 −, in humans	Blood, spleen, and inflamed joints in mice; blood, synovial fluid, and tissue in humans	ABCs were elevated in CIA mice and RA patients; In CIA mice, ABCs upregulate IFN-stimulated genes in FLS by secreting TNF-α	[103]
RA patients	CD27 − , IgD −	Peripheral blood, synovial fluid	The double-negative cell subset increased in RA patients and was associated with joint space narrowing scores and RANKL expression	[108]
Early RA patients	CD19, *ITGAX* (CD11c), *TBX21*(T-bet), *MS4A1* (CD20), *CR2* (CD21)^low^	Peripheral blood, synovial fluid	ABCs in synovial fluid were higher than in peripheral blood; Peripheral blood ABCs from early RA patients expressed chemokine receptors and adhesion molecules such as CXCR3, FcRL3, secreting high levels of IL-12 and IL-23 with low IL-10 levels	[16]
RA patients	CD19, CD11c, T-bet	Peripheral blood	ABC levels increased with disease activity but decreased after treatment; RA patients with high disease activity had elevated levels of IL-21, IFN-γ, IL-10, miR-142, and miR-146a	[109]
Tbx21^fl/fl^ Cd19^cre/+ ^mice	CD19, CD11c, T-bet	Spleen	ABCs exacerbated RA induced by EBV infection	[110]
RA patients	CD27 − , IgD − , CD11c, T-bet	Peripheral blood	The JAK1/3 inhibitor tofacitinib reduced ABC levels and alleviated systemic inflammation	[144]
RA patients	IgD − , CD27 −	Peripheral blood	TNF inhibitors and tocilizumab reduced double-negative cell levels	[122]
***SS***				
Aged female C57BL/6 mice	CD11b/c	Salivary glands	ABC accumulation within lymphocytic infiltrates in salivary glands	[125]
Female NOD.B10Sn-*H2b* pSS mice	T-bet, CD11c	Spleen	ABC expansion depended on MyD88; TLR7 expression was increased in the MZBs and ABCs; TLR7 induced the ABC differentiation, IgG, IgG2c, and IFN production, inflammatory cytokine production, and ANA secretion	[126]
Female NOD.B10Sn-*H2b* pSS mice	T-bet, CD11c	Spleen	TLR7 induced T-bet B cell expansion, splenomegaly, cervical lymphadenopathy, sialadenitis and dacryoadenitis, pulmonary and renal inflammation, and increased total and ANA-specific antibodies	[127]
***MS***				
MS patients	IgD − , CD27 − , CD11c, CD21^low^	Peripheral blood, CSF	ABCs were expanded in the peripheral blood and CSF ABCs displayed T cell stimulatory and proinflammatory functions	[133]
C57BL/6 EAE mice	T-bet, CD11c	Spleen, brain, spinal cord	ABCs were largely resident in the spleen γHV68 infection and EAE condition led to ABC expansion and affected ABC phenotype and functional capacities ABCs ameliorated EAE but deteriorated γHV68-EAE	[135]
***NMOSD***				
NMOSD patients	*ITGAX*(CD11c)^high^, *TBXA21*(T-bet)^high^	CSF, blood, and bone marrow	ABCs upregulated genes related to antigen presentation and TNF-α signaling ABCs were clustered in CSF and enhanced inflammatory activity T-bet was required for CD11c ABC generation	[138]
NMOSD patients	CD11c^high^	Peripheral blood	CD11c^high^ B cells were positively correlated with NMOSD severity, duration, and relapse CD11c^high^ B cells expressed chemokine receptors associated with migration into inflamed tissues and antigen presentation CD11c^high^ B cell frequency was positively correlated with Th1 cell frequency	[139]
***AS***				
AS patients, pSS patients	CD27 − , CD38^low^, CD21^low^, T-bet, CD11c	Peripheral blood	CD27 CD38^low^ CD21^low^ B cells were significantly elevated in both AS and pSS patients and participated in the inflammatory process CD27 CD38^low^ CD21^low^ B cells decreased the expression of T-bet and CD11c in AS patients compared to healthy controls	[143]

*ABC*, age-associated B cell; *ANA*, antinuclear antibody; *AS*, axial spondylarthritis; *CLE*, cutaneous lupus erythematosus; *CSF*, cerebrospinal fluid; *EAE*, experimental autoimmune encephalomyelitis; *FcRL5*, Fc receptor-like 5; *IFN*, interferon; *IgG*, immunoglobulin G; *MS*, multiple sclerosis; *MyD88*, myeloid differentiation factor 88; *MZB*, marginal zone B cell; *NMOSD*, neuromyelitis optica spectrum disorder; *pSS*, primary Sjögren’s syndrome; *T-bet*, T-box expressed in T cells; *Th1*, T helper-1 cell; *TLR7*, Toll-like receptor 7; *TNF-α*, tumor necrosis factor α; *γHV68*, gamma herpesvirus 68

### ABCs in SLE

#### ABCs-Related Mechanisms in SLE Pathogenesis

SLE is a chronic autoimmune disease featuring antinuclear autoantibodies, immune complex deposition, and inflammatory response, causing damage to the mucocutaneous, musculoskeletal, hematologic, and kidney systems [48, 49]. ABC accumulation was found in SLE patients and lupus mice [50]. The proportions of ABCs expressing CD11b and CD11c were significantly elevated in the spleens of elderly female C57BL/6 mice and lupus-prone NZB/WF1 mice. Further flow cytometric analysis revealed that these ABCs were positive for B220, IgM, CD5, CD11b, CD11c, CD19, and CD138 and expressed high levels of Fas, CD80, CD86, CD122, VCAM-1, and MHC-II [2]. In the peripheral blood of SLE patients, ABC-like cells were also observed with very similar characteristics to ABCs in mice, but ABCs in humans were IgD − , IgM − , and IgG [2]. Besides, IgD − CD27 − cells (DN cells) were largely increased in SLE patient blood and DN2 cells (CXCR5 − , CD21 − , CD19, CD11c) represented the majority of DN cells expanded in SLE [14]. CD11b B1 cell frequency was significantly elevated in lupus patients, and they expressed a higher level of CD86, thus facilitating the stimulation of CD4 T cells [51]. Interestingly, the circulating B cells derived from SLE patients expressed a higher level of CD11c than the dendritic cells from the same individuals and healthy donors. These CD11c B cells maintained low frequencies in the spleen and tonsils [5]. However, the critical obstacle in studying ABCs is the absence of a unified standard on the lineage-specific markers. Despite the lack of a unified standard on lineage-specific markers, ABCs are expanded in SLE patients and mice and serve as a critical mediator in SLE pathogenesis.

ABCs are considered the progenitors of autoreactive ASCs in SLE. In SLC mice lacking pre-B cell receptors, most ABCs expressed MBC markers, underwent SHM, and secreted typical lupus autoantibodies such as anti-Smith antibodies [52]. ABCs are proliferative and undergo self-renewal, giving rise to plasmablasts and germinal center (GC) B cells in vivo. In the MRL/lpr SLE model, ABCs displayed heterogeneous expression of CD11b/c, T-bet, and markers associated with plasmablasts or MBCs [1]. In lupus-prone BXD2 mice, the increased CD11c T-bet B cells caused the female-predominant production of IgG and anti-DNA autoantibodies [53]. In *Def6*/*Swap70* DKO mice, lupus was induced in females, together with ABC accumulation, which proliferated and differentiated into CD11c B cells oligoclonal, thereby promoting inflammatory responses [54]. In cutaneous lupus lesions, ABCs were recruited in response to IL-21 and TLR7/9 signaling and produced localized autoantibodies like IgG2a, IgG2b, and IgG3 [15]. An increase in T-bet CD11c B cells was observed in lupus patients and positively correlated with serum antichromatin IgG levels instead of antinuclear and anti-DNA antibodies. When transiently depleting CD11c B cells by diphtheria toxin, the serum levels of antichromatin IgG and IgG2a antibodies were significantly reduced in lupus-like mice induced by chronic graft-versus-host disease [55]. CXCR5 − CD19 CD11c DN2 cells were positively correlated with serum levels of anti-Smith-D and anti-RNP-70 antibodies in SLE patients [14]. Thus, ABCs are responsible for the secretion of diverse autoantibodies in SLE progression.

TLR signals are necessary to determine the fate of ABCs in SLE. ABCs are TLR7-driven B cell populations that regulate autoimmune and antiviral responses [56]. TLR7 duplication augmented the pathogenic role of ABCs in enhancing inflammatory response in the lungs in *Def6*/*Swap70* DKO mice [54]. The TLR7^Y264H^ variant alone was enough to cause lupus in mice. Enhanced TLR7 signaling promoted the aberrant survival of active B cells and the accumulation of CD11c ABCs and GC B cells, which could be rescued by MyD88 deficiency [26]. TLR7 and MyD88 signaling was required for ABC development and chronic TLR7 stimulation was sufficient to induce ABC accumulation and anti-Smith antibody secretion [2]. TLR7 membrane antibody blocked the negative TLR7 signaling and decreased the serum anti-DNA antibodies, thus alleviating lupus nephritis in NZB/WF1 mice [57]. These findings suggest that TLR7 signaling promotes the ABC differentiation in a MyD88-dependent manner. Unlike TLR7, TLR9 knockout mice displayed exacerbation of lupus diseases compared to wild-type MRL/lpr mice. In MRL/lpr mice with TLR9^K51E^, a TLR9 point mutant lacking ligand binding, lupus was ameliorated compared to TLR9 knockout mice, which was ligand and MyD88 independent. Moreover, the MRL/lpr mice with TLR9^P915H^, another TLR9 point mutant lacking MyD88 binding, were better shielded than TLR9^K51E^ and TLR9^WT^ mice. Furthermore, the differentiation of ABCs and plasmablasts was restrained through TLR9 signaling independent of MyD88 [27]. These results indicate that TLR9 signaling suppresses the ABC differentiation in a MyD88-independent manner.

The interplay between cytokines also controls ABC generation in SLE. It was found that the expression of IL-13 receptor α1 (IL-13Rα1) was elevated in ABCs compared to follicular B cells. Furthermore, the deletion of IL-13Rα1 suppressed ABC accumulation and their differentiation into plasmablasts in Def6/Swap70 DKO mice, which was associated with the activation of STAT6, leading to reduced autoantibody production, prolonged survival, and delayed inflammation development. Unexpectedly, IL-21 stimulation had the same effect on ABC formation as the absence of IL-13Rα1 [58]. IL-21 accumulated in lupus-like lesions and elicited CD11b B cell differentiation, whereas the ABC differentiation was slightly influenced. Besides, IL-21 stimulation significantly increased T-bet expression, ABC proliferation, and differentiation, and the IgG production was prompted when combined with TLR signals [15]. It was also supported that IL-21 stimulation induced a significantly larger population of T-bet CD11c ABCs in *Def6*/*Swap70* DKO mice, which was dependent on IRF5 [31]. IFN-γ signaling contributed to the increased glycolytic capacity of ABCs, as glycolysis restriction could inhibit ABC formation. Notably, T-bet mediated glycolysis pathway genes by inhibiting *Bcl6* expression [34]. Therefore, TLR7 and TLR9 signals and IL-21 and IFN-γ play critical roles in ABC differentiation in lupus mice.

ABCs facilitate Tfh differentiation through antigen presentation in SLE. Excessive T-bet CD11c ABCs coincided with dysregulated Tfh cells in lupus models and patients. Overabundant ABCs triggered Tfh differentiation in Ship-deficient lupus mice through antigen presentation and immunological selection. TLR signaling was crucial for ABC differentiation as MyD88 ablation blocked ABC differentiation and rescued the subsequent drawbacks in Ship-deficient lupus mice [59]. Additionally, ABCs were found to induce more robust TCR signaling than follicular B cells and preferentially promote Tfh differentiation in an antigen-dependent manner. Metformin treatment suppressed antigen uptake by ABCs, thereby reducing Tfh differentiation in Sle1/2/3 triple-congenic mice [60].

Recent research has made progress in revealing the regulatory role of ABCs in lupus. Firstly, ribonuclease A family member 2 (RNase 2) was elevated in SLE patients and highly expressed in their PBMCs. In monocytes from SLE patients, the mRNA expression of RNase 2 was correlated with IL-10 mRNA expression and RNASE2 silencing reduced the mRNA and protein level of IL-10 in vivo. Notably, IL-10 facilitated ABC differentiation in SLE patients as IL-10 restored the generation of T-bet CD11c B cells after RNASE2 silencing Therefore, the RNase2/IL-10 signaling in lupus monocytes promotes ABC formation in SLE [61]. Secondly, Fc receptor-like 5 (FcRL5) was highly expressed in ABCs, and FcRL5 overexpression exacerbated systemic autoimmunity with age in SLE models, as FcRL5 upregulation facilitated TLR signaling and destroyed B cell anergy [62]. Thirdly, a reduction in P2RY8 was observed in B cells from SLE patients lacking P2RY8 variants, which was associated with increased ABCs and ASCs and deteriorated lupus nephritis. This might be explained by the positive role of P2RY8 in promoting the adverse selection of DNA-reactive B cells and preventing the development of ASCs [63]. Finally, neutrophil dysfunction was associated with persistent autoantigen exposure and autoreactivity at the onset of SLE. Myeloperoxidase-DNA complexes were positively related to the percentages of ABCs and MBCs but negatively with naïve B cells [64].

#### ABCs-Related Prognosis, Diagnosis, and Therapy of SLE

Based on the pathogenic role of ABCs in SLE development, ABCs are theoretically believed to be a prognostic and diagnostic biomarker of SLE. GWAS studies have identified that ABCs highly expressed SLE risk genes, supporting their role in producing autoantibodies and prolonging inflammation in multi-organ injury [65]. The proportions of ABCs were elevated and positively correlated with total IgG levels in patients with typical symptoms of SLE [66]. SLE patients with increased ABCs, activated B cells, and T helper cells were prone to experience disease flares at baseline and to remain active or flare during follow-up [67]. Moreover, evidence exists that local B cell activation correlates with an ABC signature and monocyte differentiation in lupus nephritis [68]. The frequency of ABC (CD11c, CD21, and T-bet) was increased in patients with lupus nephritis, and the CD21 subset was found to be more specific under lupus nephritis [69]. However, ABCs (CD11c and CD27 −) were found in healthy European Americans positive for antinuclear autoantibody instead of their SLE patients [70]. Thus, data from larger populations is still needed to define the standard on ABC-specific markers and reveal the heterogeneity of ABCs under different populations and disease conditions.

ABCs represent promising targets for therapeutic intervention. Monoclonal antibodies that specifically target B cell surface markers are sufficient to reduce or eliminate the number of ABCs. Treatment with CD20-targeting rituximab reduced the median frequency of ABC-like (IgD − , CD27 − , CD11c, and CD21 −) B cells from 20.4 to 11.3% during early follow-up [71]. Belimumab targeting the B cell cytokine BAFF rapidly decreased the percentages of CD11c CD21 ABCs during the early follow-up, which was correlated with earlier clinical improvements. However, despite the continued reduction of ABCs, the B cells showed delayed or no responses in later developmental stages [72]. Furthermore, CAR-T cells integrate with CD19-expressing ABCs and undergo rapid activation and proliferation, releasing various cytokines and killer molecules, such as perforin and granzyme, which damage the ABC membrane and cause cell death, thus significantly reducing autoantibodies in SLE patients [73]. As these monoclonal antibodies deplete total B cells, the adaptive hormonal immunity is roughly suppressed, and the risk of severe infections is thus increased. Thus, therapeutics that precisely target ABCs are believed to be more beneficial in the future.

T-bet plays a critical role in the differentiation and pathogenesis of ABCs. Previous studies have identified T-bet as one of the transcriptional factors that drive the autoimmunity of ABCs [1, 47]. T-bet is responsible for the formation and function of IgG2a-expressing MBCs in vivo [74]. T-bet cells contribute to splenic ASC accumulation and autoantibody production, including IgM and IgG anti-dsDNA antibodies in Lyn mice [75]. Conditional deletion of T-bet in B cells impaired the GC formation and reduced the serum titers of IgG2a in SLE mice, thus improving kidney function and decreasing rapid mortality [47]. Furthermore, T-bet can integrate immune signaling with metabolic programming to drive the formation and functions of pathogenic ABCs. T-bet is demonstrated to be the downstream factor of IFN-γ and regulates the glycolysis pathway genes by inhibiting the *Bcl6* expression in ABCs [34]. *Tbx21* (encoding T-bet) ablation in B cells ameliorates metabolic imbalance in adipose tissue by reducing serum IgG2c levels and proinflammatory cytokines and macrophages [76]. When blocking the glycolysis pathway by 2-deoxy-d-glucose, the numbers and percentages of ABCs, GC B cells, and ASCs were significantly reduced, which coincided with the remarkably decreased anti-dsDNA and ANAs [34]. Surprisingly, metformin diminished the phenotypic and functional characteristics of ABCs by inhibiting antigen uptake and presentation and subsequently decreasing Tfh differentiation [60]. However, there exist ABCs that do not express T-bet, making it challenging to define ABCs and resulting in inconsistencies of ABCs described in different studies [77].

CD11b and/or CD11c cells have been recently defined as equipped with ABC-like qualities. CD11c ABCs are proposed to express autoreactive antigen receptors and thereby become a critical source of pathogenic ASCs in the development of autoimmune diseases [78]. Besides, CD11b CD11c ABCs share functional characteristics with MBCs, including expressing CD80, CD73, and PD-L2/CD273 [78, 79], implying the pathogenic role of CD11c ABCs in frequent clinical relapses of autoimmune diseases [80]. The frequency of CD11c B cells was correlated with cytokine levels, particularly the level of IL-6, as well as the number of autoimmune manifestations in patients with Down’s syndrome [81]. The long non-coding RNA XIST dysfunction that silences a subset of X-linked immune genes, such as TLR7, was involved in CD11c ABCs from female patients with SLE or COVID-19 infection [82]. CD11c B cells from SLE patients exhibited a characteristic increased PD-1 and PD-L1 expression [83]. Notably, there was no correlation between CD11c B cell and SLE patients’ age [5, 83]. Depleting CD11c B cells without influencing CD11c myeloid cells ameliorated lupus disease [1]. These results support that targeting CD11c ABCs may be an effective therapeutic strategy for SLE.

CD11b plays a crucial role in regulating proinflammatory TLR signaling. The single nucleotide polymorphisms of the *ITGAM* gene (encoding CD11b) are associated with increased risk for SLE and lupus nephritis as they lead to defective integrin with reduced binding affinity, blocked cell adhesion and phagocytosis, and reduced capability to inhibit inflammatory cytokine production [84]. CD11b agonist LA1 partially activated integrin, reduced IFN response, and protected lupus-prone MRL/Lpr mice. CD11b activation inhibited IFN signaling and TLR-induced proinflammatory response through AKT/FoxO3/IRF3/7 pathway [85]. Besides, CD11b B1 cell frequency was markedly elevated in lupus patients, and these cells secreted modest levels of antibodies and enhanced T-cell stimulatory activity through CD86 [51]. CD11b-deficient B cells produced lower levels of IgG1 and IgG2a in response to LPS stimulation, and the class-switch recombination and affinity maturation of antibodies were profoundly inhibited in mice lacking CD11b [86]. Intriguingly, autoreactive B cells lacking CD11b were hyperproliferative and prolonged survival in response to BCR crosslinking. The autoantibody production and immune complex deposition in the kidney were enhanced in CD11b-deficient mice with BCR engagement [87]. Recently, GB1275 has been identified as a first-in-class CD11b agonist, which regulates interferon gene expression through the STING/STAT1 pathway [88]. Therefore, CD11b plays a key role in regulating the inflammatory response and antibody production, and exogenous CD11b activation in ABCs can be a potential therapeutic strategy for SLE.

The concentration of adenosine that mitigates the hyperactivity of the human immune system was significantly decreased in the serum of SLE patients [89]. Meanwhile, the expression level of A2AR receptors (A2AR) negatively correlated with the SLE disease activity [90]. Compared to CD11c − B cells, the expression of A2AR was approximately ten times higher in CD11c T-bet B cells [91]. The activation of A2AR enhanced the generation of regulatory T cells, inhibited the proliferation of effector T cells and Tfh cells, and blocked the formation of GC B cells, thus suppressing proinflammatory cytokine production [92–96]. Treatment with an A2AR agonist reduced the level of serum anti-dsDNA and renal immune complex deposition in MRL/lpr-induced lupus nephritis mice [97]. Moreover, the A2AR agonist could deplete CD11c T-bet B cells, thus reducing ANA in lupus-prone mice and alleviating renal pathology [98]. Therefore, A2AR activation is a promising immunosuppressive target in autoimmune diseases.

### ABCs in RA

RA is a systemic autoimmune disease featuring chronic inflammation and cartilage and bone erosion in affected joints [99]. The RA pathophysiology involves the breakdown of immune tolerance, resulting in immune cell infiltration in the synovium, autoantibody production, such as rheumatoid factor (RF) and anticitrullinated protein antibody (ACPA), chronic inflammation, and subsequent cartilage and bone tissue destruction. This ultimately leads to joint deformation and functional loss [100]. ABCs contribute to RA pathogenesis possibly by secreting autoantibodies and proinflammatory cytokines, promoting antigen presentation and T-cell activation, and interacting with FLS [11].

By integrating single-cell transcriptomics with mass cytometry, Zhang et al. first revealed aberrant expansion of ABCs in RA joint synovial tissues, suggesting their potential role in RA [101]. In RA patients, ABCs highly express T-bet and co-express CD11c and CD19 while lacking CD27 and CD21 [102]. Additionally, they are characterized by IgD − , IgM − , IgG − , CD38^low^, CD5^high^, CD80^high^, CD86^high^, CD20^high^, and CD23 expression profiles [2]. The expression of IL-13Rα1, an IL-4 ligand, was higher in ABCs than in CD11c − B cells. As for ASC markers, no significant increase in BCMA/CD269 or CD138 expression was observed [103]. These results reveal ABCs’ unique phenotypic characteristics in RA.

In RA patients, the levels of CCR2 and its ligand CCL2 in synovial tissue and serum were significantly elevated and correlated with disease activity [104]. Notably, CCR2 expression was upregulated in ABCs [103]. ABCs from early RA patient blood expressed CXCR3 [16], which was abundant in arthritic joints [105]. By binding to ligands such as CXCL9, CXCL10, and CXCL11 highly expressed in synovial fluid [106], CXCR3 induced the migration of activated T cells to inflammatory sites [105]. Thus, the high expression of CCR2 and CXCR3 may stimulate ABCs to migrate and infiltrate synovium, exacerbating the inflammatory response in joints [107]. These results suggest that chemokines and their receptors are important in ABC migration in RA.

Increased ABC levels were observed in the blood, spleen, and inflamed joints of collagen-induced arthritis (CIA) mice and the blood and synovial fluid and tissue in RA patients [103]. ABCs are the major B cell subsets in synovial fluid, numerically distinct from those in peripheral blood [16]. ABCs, also known as IgD − CD21 − B cells, were associated with the joint space narrowing score and the expression of RANKL [108]. In PBMCs from RA patients, the proportion of ABCs increased with higher disease activity but decreased in response to treatment [109]. Intriguingly, the RA exacerbation induced by Epstein-Barr virus (EBV) infection was alleviated in ABC-knockout mice [110]. Targeting T-bet-expressing ABCs has proven effective in improving the general well-being of patients with various autoimmune diseases, including RA [10]. Therefore, ABCs participate in RA pathogenesis and are crucial therapeutic intervention targets.

The generation of autoantibodies, mainly RF and ACPA, is a hallmark of RA [111]. The percentage of ABCs was correlated with proportions of Tfh cells and plasma IL-21, miR-142, and miR-146a, which jointly drove the differentiation from naïve B cells into ABCs [109]. Through interacting with Tfh cells, ABCs underwent somatic hypermutation, isotype switching, and avidity maturation, ultimately developing into ASCs that may produce RF and ACPA [112]. These autoantibodies induced the formation of immune complexes in synovium, triggering complement activation, leukocyte infiltration, and autoreactive antigen presentation, thereby aggravating inflammation [113]. Additionally, ACPA directly interacted with osteoclast precursors, inducing osteoclast differentiation and bone resorption [114].

ABCs display a complex cytokine profile distinct from other B cell subpopulations in RA, secreting proinflammatory cytokines like IL-21, IL-17A, TNF-α, and IFN-γ [103, 115], as well as regulatory cytokines such as IL-4 and IL-10 that suppress inflammation [116, 117]. Among them, IL-21, IL-17A, and IFN-γ triggered the abnormal JAK/STAT signaling in the synovium, leading to joint destruction [118], while TNF-α activated the NF-κB pathway, enhancing TNFR-II expression and stimulating RANKL secretion in RA-FLS, thereby facilitating osteoclastogenesis [103]. Besides, the levels of TNF and IL-6 expression were significantly elevated in ABCs derived from old mice compared to those from young mice [119]. Intriguingly, TNF-α secreted by ABCs from old mice could induce apoptosis and inhibit the growth of B-cell precursors, contributing to the loss of B-cell precursors in the bone marrow of old mice. These TNFα-mediated effects could be ameliorated by the anti-inflammatory IL-10 secreted by splenic and circulating follicular B cells [115]. The proportion of ABCs was positively correlated with Tfh cells and plasma levels of IL-21 [28]. ABCs preferentially secreted IL-4 and IL-10 upon TLR7 or TLR9 stimulation in vitro [4]. ABCs derived from early RA patient blood produced high levels of IL-12 and IL-23, but low levels of IL-10 [16]. Moreover, T-bet-expressing marginal zone B cells were able to attenuate CIA by promoting the regulatory capacity of IL-10 [120]. These findings indicate that ABC-derived cytokines play a complicated role in RA progression.

ABCs have been implicated in inducing the activation of FLS, which are critical drivers of inflammation in RA. When coculturing ABCs with FLS derived from RA patients, RA ABCs upregulated chemotaxis-related genes compared to naïve B cells and MBCs [103]. Concurrently, FLS facilitated the migration of ABCs beneath the synoviocytes, dependent on SDF-1 and VCAM-1, fostering ABCs-FLS interaction that further shifted FLS to a proinflammatory phenotype [121]. In CIA mice, ABCs-derived TNF-α upregulated IFN-activated genes in FLS through the ERK1/2 and JAK-STAT1 pathways. This activation of FLS increased IL-6, MMP-1, MMP-3, and MMP-13 levels, sustaining inflammation and leading to cartilage destruction [103]. Thus, the cytokine interplay and cellular interactions between ABCs and FLS are critical to RA pathogenesis.

Specifically targeting ABCs holds promise as a therapeutic strategy for RA. Therapeutic strategies for RA are like those for SLE, such as blocking surface markers, such as CD19 and CD11b/c, and inhibiting activation-related cytokines like IL-21 and IFN, as well as TLR and JAK/STAT signaling. Additionally, TNF inhibitors and tocilizumab, an IL-6 receptor antagonist, have been proven to reduce the frequency of ABCs/DN B cells without affecting other B cell subpopulations [122]. Notably, the upregulation of A2AR exhibited an inverse correlation with disease activity, suppressing the release of inflammatory cytokines, such as TNF-α, IL-1β, and IL-6, and metalloproteinases like MMP-1 and MMP-2 [95, 96]. Given the high expression of A2AR in T-bet CD11c B cells [96], the efficacy of A2AR agonist treatment has recently been demonstrated in lupus-prone mice [98], suggesting that A2AR may be a novel therapeutic target in RA.

### ABCs in Other Autoimmune Diseases

SS is a chronic autoimmune disease featuring lymphocytic infiltration in the salivary and lacrimal glands, causing progressive glandular dysfunction and resultant xerostomia and xerophthalmia [123]. Autoreactive ABCs and ASCs producing autoantibodies accounted for the disease progression [124]. In pSS, ABCs were enriched in salivary glands compared to the spleen or other organs and were correlated with increased Tfh cells. Subsequently, lymphocytic infiltration developed in the salivary glands, and then levels of serum autoantibodies increased in aged mice [125]. ABC expansion was observed in the pSS mouse model, with enhanced expression of TLR7 and increased sensitivity to TLR7 agonists in a MyD88-dependent manner. TLR7-activated murine ABCs and marginal zone B cells enhanced inflammatory cytokine secretion and antinuclear autoantibody production [126]. TLR7 agonist imiquimod treatment resulted in pronounced lymphocytic infiltration of exocrine tissues, kidneys and lungs, salivary hypofunction and elevated levels of antinuclear autoantibodies, including Ro and La antibodies. In addition, TLR7 activation promoted T-bet B cell expansion and accelerated disease progression in pSS mice [127]. Therefore, therapeutics targeting TLR7 signaling in B cells may offer promising outcomes for treating SS. In an open-label trial of 30 patients with pSS, the use of belimumab, a monoclonal antibody against BAFF to deplete ABCs, enabled 60% of participants to achieve the primary endpoint with less dryness, fatigue and pain VAS scores [124, 128]. Moreover, the administration of telitacicept, which blocks both BAFF and proliferation-inducing ligand (APRIL), prominently reduced the ESSDAI and MFI-20 scores and serum levels of immunoglobins [129]. Additionally, BTK blockage inhibits the IL-21 signaling by decreasing the phosphorylation level of STAT1, thus inhibiting the pathogenicity of CD11c ABCs [130, 131]. Providing the critical role of CD40-CD40L interaction in ABC generation, the anti-CD40 antibody iscalimab may be useful in SS treatment. Therefore, targeting CD11c ABCs is an attractive approach for SS treatment.

MS is a chronic autoimmune disease of the central nervous system featuring inflammatory demyelination and neuronal injury [132]. Few studies have focused on the role of ABCs in MS progression. A higher proportion of ABCs, including IgD − CD27 − and CD11c CD21 − B cells, has been observed at the periphery of MS patients. Besides, the frequency of ABCs increases in the cerebrospinal fluid of MS patients, where they induce T-cell responses and produce proinflammatory cytokines, thereby exacerbating neuroinflammation [133]. Strong evidence suggests that EBV-induced autoimmunity increases the risk of MS, while ABCs are critical in this process [12, 134]. The proportion of circulating ABCs was elevated by EBV infection and relapse-remitting MS conditions. Besides, EBV infection non-significantly elevated the percentage of Fas-expressing ABCs, which was significantly decreased in relapse-remitting MS patients [135]. Notably, the phenotype of ABCs differs between γHV68-infected MS and EAE mouse models. Specifically, γHV68 infection increases IFN-γ-expressing ABCs, whereas EAE increases IL-17A-expressing ABCs [135]. Recent evidence indicates ABCs may participate in inhibiting the reactivation of γHV68 during heterologous infection [136]. However, more studies are needed to reveal the mechanism of ABCs in EBV-induced MS aggravation.

NMOSD is an acute or subacute autoimmune disease featuring acute optic neuritis and transverse myelitis, resulting from pathogenetic IgG autoantibodies against aquaporin 4 (AQP-4) or myelin oligodendrocyte glycoprotein [137]. ABCs were identified as significant B cell subpopulations in the cerebrospinal fluid, blood, and bone marrow of NMOSD patients [138]. The frequency of CD11c^high^ B cells was significantly elevated in NMOSD patients, which was associated with increased Tfh cell frequency. Furthermore, high levels of CD11c^high^ B cells were positively correlated with disease severity, duration, and relapse [139]. The NMOSD B cells enhanced proinflammatory activity and gene expression of chemokine receptors, such as CXCR3 and CXCR4, and became hyperresponsive to IFN-α/β, facilitating B cell maturation and AQP-4 autoantibody production. Therefore, ABCs may be involved in NMOSD development, but more research is warranted.

AS is a chronic inflammatory disease, primarily impacting the axial skeleton, featuring chronic back pain, spinal stiffness, and peripheral and extra-musculoskeletal manifestations [140]. AS is an autoimmune disease evidenced by T-cell activation and expansion and autoantibody presence, including anti-CD74, anti-sclerostin, and anti-noggin antibodies [141]. Emerging evidence supports that B cells participate in AS pathogenesis. Disturbances in circulating B cell populations, B cell infiltration, and autoantibody formation in the axial skeleton have been observed in AS patients. Rituximab-induced B cell depletion has shown beneficial effects in AS patients [142]. However, the proportions of CD27 CD38^low^ CD21^low^ B cells were increased while the proportions of T-bet and CD11c-expressing CD27 CD38^low^ CD21^low^ B cells were decreased in AS patients. This suggested that T-bet and CD11c-expressing CD27 CD38^low^ CD21^low^ B cells might serve as precursor cells for ASCs or migrate to the inflammatory sites in AS pathogenesis. Additionally, single nucleotide polymorphisms in the *TBX21* gene encoding T-bet were associated with AS [143]. Therefore, the precise role of ABCs in AS pathogenesis remains largely unclear.

## Conclusions and Future Perspectives

In summary, ABCs are a heterogeneous subpopulation of B cells with distinct transcription profiles, extrafollicular differentiation processes, and multiple functions in autoimmunity. ABCs can proliferate and differentiate upon TLR7/9 signal activation under the combined regulation of cytokines including IFN-γ and IL-21, chemokine receptors including CXCR3 and CCR2, and integrins including CD11b and CD11c. ABCs uptake and present antigens, produce autoantibodies and proinflammatory cytokines, and activate T helper cells in the development of autoimmune diseases. As the proportion of ABCs is positively correlated with disease activity in SLE and RA patients, ABCs are potential prognostic and diagnostic biomarkers. Specifically targeting ABCs by inhibiting T-bet and CD11c and activating CD11b and ARA2 represents potential therapeutic strategies for SLE and RA.

The scRNA-seq technology has shown enormous potential in revealing the heterogeneous characteristics of ABCs across different diseases and patients. Recent findings from the scRNA-seq analysis show that ABC differentially expressed genes encoding transcription factors, cytokines, and signaling molecules compared to other B cell subpopulations [145]. Notably, autoimmune regulator (AIRE) was significantly upregulated in classical ABCs, the frequency of which was elevated in females and SLE patients [146]. AIRE controls the negative selection of autoreactive B cells to maintain peripheral tolerance [147]. However, the function of AIRE has not been accessed in ABCs. By single-cell RNA-sequencing and flow cytometry, CD11b CD11c T-bet ABCs were identified in atherosclerosis for the first time [45]. Single-cell gene expression and BCR sequencing data from synovial B cells showed that DN2 B cells are the primary precursors of synovial ASCs in RA [148]. In lupus nephritis, ABCs were recognized by scRNA-seq, and the intermediate states between naive B cells and ABCs were revealed by trajectory analysis [68]. scRNA-seq and VDJ analysis have suggested that ABCs are profoundly dynamic, chronically reactivated, and share clonal mutations with the plasmablasts [1]. Besides, scRNA-seq also helps to identify novel transcription factors like ZEB2, which controls lineage specification and defines the cellular identity of ABCs [144]. scRNA-seq is expected to identify new intermediate subpopulations, new transcription regulators, and membrane surface markers during ABC differentiation and describe the distinct stages by which B cells migrate and become ABCs.

The gene expression profile and genetic regulation of ABCs support the pathogenic role of ABCs in autoimmune diseases. Vinuesa CG and colleagues compared expression data of SLE-associated genes by integrating information from GWAS. They found that ABCs expressed high levels of typical *TBX21* (encoding T-bet), *ITGAX* (encoding CD11c), *ITGAM* (encoding CD11b), *FCRL2*, *FCRL5*, *FCRL3*, *TLR7,* and *ZEB2* [65]. Notably, ZEB2 is essential for ABC differentiation by targeting genes related to ABC specification and function, such as *ITGAX*, dependent on the JAK/STAT signaling pathway [144]. ABCs from early RA patients also expressed high levels of *CD19*, *ITGAX*, *TBX21*, and *CD20*, but low levels of *CD21*. Aside from typical ABC markers, ABCs displayed high expression of genes associated with apoptosis (*Fas*, *CASP1,* and *CASP8*), plasma cell differentiation (*PRDM1* and *XBP1*), class-switch recombination and somatic hypermutation (*AICDA*), adhesion molecules (*CD97*), inflammatory chemokine receptor (*CXCR3* and *CX3CR1*). Moreover, ABCs had high expression of *TNF* and *IL12A* and low expression of *IL23A*, *IL6,* and *IL6ST* (encoding gp130). Additionally, ABCs showed elevated expression of inhibitory receptors (*FCGR2A/C*, *FCGR2B,* and *LILRB1-3*) and immunomodulatory molecules (*TLR9*, *CD80,* and *CD86*) [16]. In individuals exposed to chronic malaria, ABCs upregulated *TBX21*, *ITGAX* , *FCRL5*, *ITGB2*, *CD72A*, *CD63*, *CD84,* indicating that ABC shared similar transcriptional profiles in malaria and autoimmune diseases [145].

The X-linked *TLR7* gene may be a key factor in the sex differences affecting the frequency and function of ABCs [149]. In mammals, females have one extra X chromosome, which is often inactivated to prevent gene expression imbalances [150]. *TLR7* has recently been shown to partially escape from X-inactivation and resultantly overexpress in females [151]. In *Def6*/*Swap7* DKO female mice, ABC accumulation is more significant than in age-matched male mice. Partially translocating the X chromosome to the Y chromosome in *Def6*/*Swap7* DKO mice (Yaa-Def6/Swap7 DKO) leads to increased *TLR7* expression and more splenic ABCs, thus aggravating autoimmune diseases. Moreover, ABCs expressed different levels of IFN-stimulated genes in female and male mice. ABCs in Yaa-*Def6*/*Swap7* DKO male mice are enriched for *IFNα* and *IFNγ*, whereas ABCs in *Def6/Swap7* DKO male mice upregulate genes associated with hemostasis and platelet activation. There is also a sex difference in the ability of ABCs to differentiate into distinct effector subsets. For example, *Def6/Swap7* DKO female mice exhibit a stronger expansion of CD11c GC B cells and plasmablasts/ASCs while male mice are more prone to generate CD11c− subsets. TLR7 regulates the generation of CD11c or CD11c− effector subsets from ABCs by inhibiting ROCK2 phosphorylation and directly affecting IRF8 activity [54].

ABCs may serve as an emerging and promising therapeutic target. Specifically, based on ZEB2-mediated transcription, the JAK-STAT signaling is essential for the differentiation and pathogenicity of ABCs, indicating that JAK inhibitors are a promising therapy. Baricitinib, a JAK1/2 inhibitor, or tofacitinib, a JAK1/3 inhibitor, significantly reduced ABC frequency in vitro [144]. For SLE patients with excessive TLR7 activation and extrafollicular ABC accumulation, inhibiting TLRs and downstream MyD88 might be a therapeutic approach in SLE and lupus nephritis [26, 152]. Besides, inhibiting IFN is an effective way to target T-bet ABCs, as anifrolumab inhibits the interaction between the interferon receptor and ligands in SLE, showing positive outcomes in a phase III trial [153]. Considering the important regulatory role of IRF5 for ABCs, indirect targeting of ABCs may be achievable through IRF5 inhibitors. Genetic and chemical inhibition of IRF5 effectively alleviated preexisting lupus-like syndrome in mice [154]. Although ABCs provide novel multiple targets for potential interventions, further investigations are needed to validate the therapeutic efficacy in treating various autoimmune diseases.

## Data Availability

No data was used for the research described in the article.
